# Precision spectroscopy on ^9^Be overcomes limitations from nuclear structure

**DOI:** 10.1038/s41586-024-07795-1

**Published:** 2024-08-14

**Authors:** Stefan Dickopf, Bastian Sikora, Annabelle Kaiser, Marius Müller, Stefan Ulmer, Vladimir A. Yerokhin, Zoltán Harman, Christoph H. Keitel, Andreas Mooser, Klaus Blaum

**Affiliations:** 1 Max Planck Institute for Nuclear Physics, Heidelberg, Germany; 2 Institute for Experimental Physics, Heinrich Heine University Düsseldorf, Düsseldorf, Germany; 3 Ulmer Fundamental Symmetries Laboratory, RIKEN, Saitama, Japan

**Keywords:** Atomic and molecular physics, Quantum physics

## Abstract

Many powerful tests of the standard model of particle physics and searches for new physics with precision atomic spectroscopy are hindered by our lack of knowledge of nuclear properties. Ideally, these properties may be derived from precise measurements of the most sensitive and theoretically best-understood observables, often found in hydrogen-like systems. Although these measurements are abundant for the electric properties of nuclei, they are scarce for the magnetic properties, and precise experimental results are limited to the lightest of nuclei^1–4^. Here we focus on ^9^Be, which offers the unique possibility to use comparisons between different charge states available for high-precision spectroscopy in Penning traps to test theoretical calculations typically obscured by nuclear structure. In particular, we perform high-precision spectroscopy of the 1*s* hyperfine and Zeeman structure in hydrogen-like ^9^Be^3+^. We determine the effective Zemach radius with an uncertainty of 500 ppm, and the bare nuclear magnetic moment with an uncertainty of 0.6 parts per billion— uncertainties unmatched beyond hydrogen. Moreover, we compare our measurements with the measurements conducted on the three-electron charge state ^9^Be^+^ (ref. ^5^), which enables testing the calculation of multi-electron diamagnetic shielding effects of the nuclear magnetic moment at the parts per billion level. Furthermore, we test the quantum electrodynamics methods used for the calculation of the hyperfine splitting. Our results serve as a crucial benchmark for transferring high-precision results of nuclear magnetic properties across different electronic configurations.

## Abstract (web summary)

A high-precision measurement of the shielding effects of multiple electrons on the nuclear magnetic moment is made possible by spectroscopy of hydrogen-like ^9^Be^3+^, which overcomes limitations from the nuclear structure.

## Main

Historically, advances in precision atomic spectroscopy have progressed alongside the development of the quantum theories of nature to successfully explain even the smallest of contributions to atomic transition frequencies. Meanwhile, precise tests and searches for new physics require that the established theories provide sufficiently accurate predictions for the observed system. Nowadays, in atomic systems, this frequently necessitates the knowledge of nuclear properties. However, these properties are often not known accurately enough from theoretical models describing the nuclear structure and are instead determined experimentally from independent measurements with high sensitivity to nuclear structure. For example, measurements of the Lamb shift in atomic^6^ or muonic hydrogen^7^ and deuterium^8^ are combined with other transitions to independently determine the proton charge radius and the Rydberg constant^9^. However, discrepancies between competing results remain unsolved^10^. Likewise, the magnetic dipole–dipole interaction of the nucleus with the bound electrons, which results in the hyperfine splitting (HFS), depends on the magnetic moment of the nucleus and is markedly influenced by the Zemach radius—a measure of electric and magnetic form factors of the nucleus. Here measurements of the 1*s*-HFS interval in hydrogen-like systems are the most sensitive to the Zemach radius and serve as ideal references to evaluate the nuclear structure effects in other HFS intervals and test the quantum electrodynamics (QED)^11^. However, for low nuclear charge *Z*, these measurements exist only for the hydrogen isotopes^11^ and ^3^He (refs. ^4,12^), whereas for high *Z*, tests of the HFS lack accurate experimental values of the nuclear magnetic moments^13–15^.

Recently, the high-precision Penning-trap measurement of the Zeeman and hyperfine splitting of ^3^He^+^ allowed us to directly measure its Zemach radius and nuclear magnetic moment^4^, simultaneously providing both parameters needed for precise predictions of other HFS intervals^12^. Moreover, the accurate value of the magnetic moment of the atom enables absolute magnetometry with hyperpolarized ^3^He (ref. ^16^). However, this requires transferring the measured nuclear magnetic moment from the hydrogen-like system to the neutral system, which involves the theoretical calculation of diamagnetic shielding parameters. In the past, inadequate calculations of these parameters have led to several discrepancies in precision physics^13,17–19^, including the recent 7*σ* deviation of the HFS specific difference in ^209^Bi^82+,80+^. In these studies, the required nuclear magnetic moments were obtained from measurements using systems with complex electronic structures and calculations of shielding parameters relying on quantum chemistry codes that frequently provide no, or underestimated, uncertainties^15^. By contrast, for systems such as hydrogen-like and neutral ^3^He, these issues are remedied by the simple electronic structure, which enables diamagnetic shielding calculations using highly accurate non-relativistic quantum electrodynamics methods. Here the diamagnetic shielding parameters are calculated in a perturbative approach, and the estimated uncertainties are more than one order of magnitude better than the experimental value of the ^3^He nuclear magnetic moment^20^. However, adjustments to the non-relativistic quantum electrodynamics theory value at the same level as the experimental uncertainty were performed recently^21^, further motivating an experimental verification and benchmark for diamagnetic shielding calculations.

An ideal candidate to both test the diamagnetic shielding calculations and introduce a highly accurate reference for nuclear structure contributions in the hyperfine interaction is ^9^Be. Here the low nuclear charge of *Z* = 4 permits calculations of the highest available accuracy in the hydrogen-like system, while, simultaneously, the Zeeman and hyperfine splitting can be probed using high-precision spectroscopy in Penning traps for both the lithium-like ^9^Be^+^ and hydrogen-like ^9^Be^3+^ charge states. In this work, we present the first measurements, to our knowledge, on ^9^Be^3+^. Compared with extractions using ^9^Be^+^ (refs. ^5,22^), the higher accuracy of theoretical calculations possible in hydrogen-like ^9^Be^3+^ allows for markedly improved determinations of the Zemach radius as well as the magnetic moment of the bare nucleus. Furthermore, we perform a unique comparison between the experimental hyperfine and Zeeman splitting of ^9^Be^+^ and ^9^Be^3+^, which we use to eliminate the nuclear structure-dependent terms. Compared with the measurements of the hyperfine splittings of ^209^Bi^82+,80+^ (ref. ^13^), this allows not only testing the QED theory using the HFS specific difference but also testing calculations of multi-electron diamagnetic shielding parameters at the parts per billion (ppb) level. The latter constitutes the first precision test of the corrections to nuclear magnetic moments across different charge states.

**Fig. 1:**
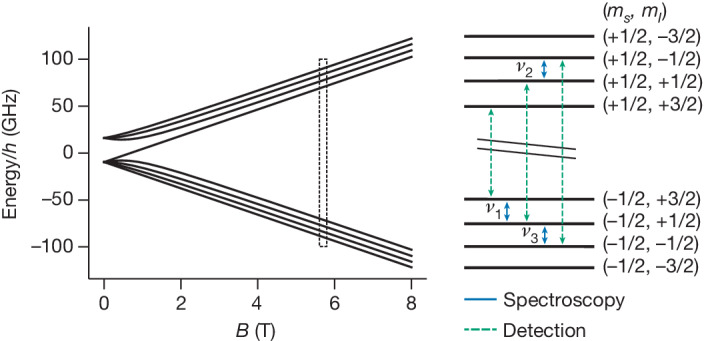
Hyperfine and Zeeman splitting of ^9^Be^3+^. On the left, the energies of the spin states are shown as a function of the external magnetic field *B*. The level structure at our magnetic field (dashed box) of around 5.7 T is shown on the right. Spectroscopy is performed on the three nuclear spin transitions (blue) labelled *ν*_1_, *ν*_2_ and *ν*_3_. The three electron spin transitions (green) are used for detecting the nuclear spin state.

The combined hyperfine and Zeeman interaction in ^9^Be^3+^ is described by the Hamiltonian

1 $$H=-\frac{1}{2{\rm{\pi }}}\frac{e}{2{m}_{{\rm{e}}}}{g}_{s}B{S}_{z}-\frac{1}{2{\rm{\pi }}}\frac{e}{2{m}_{{\rm{p}}}}{g}_{I}^{{\prime} }B{I}_{z}+{\nu }_{{\rm{HFS}}}{\bf{S}}\cdot {\bf{I}},$$

where *e* is the elementary charge; *m*_e_ and *m*_p_ are the electron and proton masses, respectively; and *ν*_HFS_ is the hyperfine splitting. The external magnetic field *B* is chosen to define the *z*-direction as the quantization axis for the spin angular momenta of the electron **S** and nucleus **I**. In this formula, the magnetic moments of the bound electron and shielded nucleus are given in units of the Bohr and nuclear magneton using the gyromagnetic ratios (*g*-factors), *g*_*s*_ and *g*′_*I*_ (the prime indicates the shielding), respectively. Using the spin magnetic quantum numbers of the electron, *m*_*s*_, and the nucleus, *m*_*I*_, the level structure of ^9^Be^3+^ is shown in Fig. 1. The 12 magnetic dipole transitions can be split into six low-frequency, 5.7 GHz < *ν* < 7.2 GHz, nuclear spin transitions with (Δ*m*_*s*_, Δ*m*_*I*_) = (0, 1); four high-frequency, 141 GHz < *ν* < 181 GHz, electron spin transitions with (Δ*m*_*s*_, Δ*m*_*I*_) = (1, 0); and two high-frequency combined transitions with (Δ*m*_*s*_, Δ*m*_*I*_) = (1, 2). The highest sensitivity for the extraction of *g*′_*I*_ and *ν*_HFS_ is reached with the measurement of the two nuclear transitions *ν*_1_ ≈ 6.622 GHz and *ν*_2_ ≈ 6.553 GHz (compare Fig. 1). Our determination of the magnetic field *B* requires the precise knowledge of the mass ${m}_{{}^{9}{\rm{Be}}^{3+}}$ of the ^9^Be^3+^ ion. As the uncertainty $\delta {m}_{\genfrac{}{}{0ex}{}{9}{}{\rm{Be}}}/{m}_{\genfrac{}{}{0ex}{}{9}{}{\rm{Be}}}=9\times 1{0}^{-9}$ of the current accepted mass value^23^ would limit the extraction of *g*′_*I*_, the measurement of a third nuclear transition *ν*_3_ ≈ 6.124 GHz was included, allowing us to independently determine the mass.

**Fig. 2:**
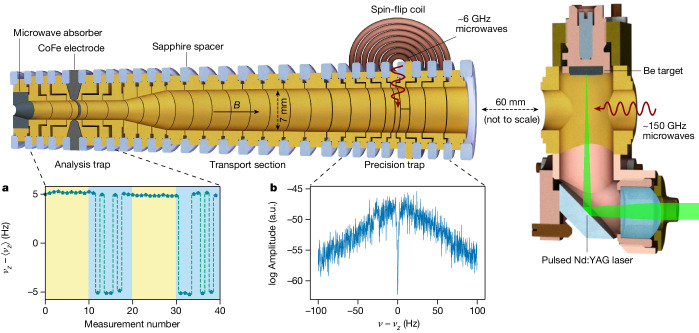
Schematic of the Penning-trap setup. The Penning trap is built up of gold-plated copper electrodes separated by isolating sapphire spacers (blue). Microwaves for driving the electron spin transitions are irradiated on-axis by a waveguide, and a coil with a few windings connected to coaxial cables is used to drive the low-frequency nuclear transitions in the precision trap. ^9^Be^3+^ ions are produced using laser ablation from a solid beryllium target and subsequent electron impact ionization. **a**, While alternating the irradiation of the two electron spin detection transitions (indicated by the two background colours) in the analysis trap, axial frequency jumps are observed only for one of them. **b**, The motional frequencies of ^9^Be^3+^ are measured by dip signals in the Fourier spectrum of the detection signal. a.u., arbitrary units.

The measurements are performed with a single ion in the Penning-trap setup shown in Fig. 2. The setup is placed in a sealed-off vacuum chamber inside the liquid-helium-cooled bore of a 5.7-T superconducting magnet. Inside this chamber, the vacuum conditions allow for trapping lifetimes exceeding the multiple months needed for the full measurement campaign. The magnetic field confines the ion on a circular orbit with revolution frequency ${\nu }_{c}=(qB)/(2{\rm{\pi }}{m}_{{}^{9}{\rm{Be}}^{3+}})$, where *ν*_c_ is the cyclotron frequency and *q* = 3*e* is the charge of ^9^Be^3+^. The cylindrical electrodes of the trap are biased by an ultrastable voltage source to create a quadrupolar electrostatic potential, forcing the ion into a harmonic oscillation along the *z*-axis with frequency *ν*_*z*_. This further splits the radial motion into two eigenmotions characterized by the modified cyclotron frequency *ν*_+_ and the magnetron frequency *ν*_−_. Here *ν*_c_ ≈ *ν*_+_ ≈ 29 MHz ≫ *ν*_*z*_ ≈ 480 kHz ≫ *ν*_−_ ≈ 4 kHz. By measuring the three eigenfrequencies and combining them using the so-called invariance theorem, ${\nu }_{{\rm{c}}}^{2}={\nu }_{+}^{2}+{\nu }_{-}^{2}+{\nu }_{z}^{2}$, the cyclotron frequency is reproduced while cancelling certain systematic effects^24^. A superconducting tank circuit is connected to one of the trap electrodes to provide resistive cooling of the axial mode to the ambient 4.2 K as well as detection of the axial oscillation^25^ (Fig. 2). Sideband coupling to the axial mode enables thermalization and frequency measurement of the radial modes^26^.

For the spectroscopy of the spin transitions, we count spin-state changes of the ion following an excitation with a frequency close to the transition centre. Changes in the spin state are detected by using the continuous Stern–Gerlach effect^27^. To this end, a ferromagnetic ring electrode introduces a quadratic magnetic field Δ*B* = *B*_2_*z*^2^, where *B*_2_ ≈ 282 kT m^−2^. This couples the magnetic moment of the ion to its axial motion, slightly altering *ν*_*z*_ depending on the spin state. Changes in the spin state induced by a transition (*m*_*s*_, *m*_*I*_) → (*m*′_*s*_, *m*′_*I*_) lead to a shift Δ*ν*_*z*_ proportional to the change of the magnetic moment^27^. For an electron spin transition, (Δ*m*_*s*_, Δ*m*_*I*_) = (1, 0), the axial frequency jump Δ*ν*_*z*_ ≈ 10 Hz can be easily detected. By contrast, the change of the magnetic moment of the ion for a nuclear spin transition, (Δ*m*_*s*_, Δ*m*_*I*_) = (0, 1), is greatly reduced, making its detection challenging. For instance, in the case of *ν*_1_, the axial frequency jump is only Δ*ν*_*z*_ ≈ 6 mHz, which cannot be discerned from the background fluctuations of *ν*_*z*_. Instead, for the spectroscopy of nuclear transitions (*m*_*s*_, *m*_*I*_) → (*m*_*s*_, *m*′_*I*_), the two detection transitions (*m*_*s*_, *m*_*I*_) → (*m*′_*s*_, *m*_*I*_) and (*m*_*s*_, *m*′_*I*_) → (*m*′_*s*_, *m*′_*I*_) are used, compare Fig. 1. While cycling these two transitions, only one of them produces detectable electron spin-state changes (Fig. 2a), which unambiguously identifies the nuclear spin state.

As the large *B*_2_ required for spin-state detection would limit the experimental precision, we use spatially separated traps for spin-state detection and the precision measurement, called analysis trap (AT) and precision trap (PT) (ref. ^28^) (Fig. 2). In the PT, the residual magnetic field inhomogeneity is greatly reduced, *B*_2,PT_ ≈ 1 T m^−2^. A measurement cycle starts by determining the spin state in the AT. Following an adiabatic transport to the PT, an initial cyclotron frequency measurement *ν*_c,1_ determines the expected spin transition frequency. During a second measurement of the cyclotron frequency *ν*_c,2_, the spin transition is driven with a frequency *ν*_MW_, which is randomly offset from the previously calculated value. After a third measurement, *ν*_c,3_, the ion is transported back to the AT to again detect the spin state and determine whether it changed from the previously detected one. We measured *ν*_c_ to a precision of one part in a billion with typical averaging times of a few minutes and performed a single measurement cycle in 20 min. For each of the three transitions, a few hundred measurement cycles were performed.

**Fig. 3:**
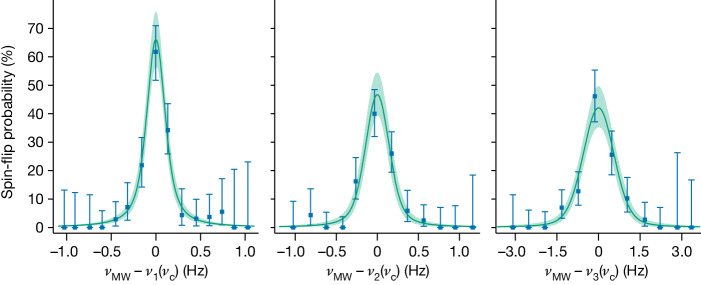
Recorded resonance curves. The difference of the probe excitation at frequency *ν*_MW_ to the nuclear spin transition frequency *ν*_*i*_(*ν*_c_) is calculated from the simultaneously measured *ν*_c_. The data points are the binned number of successful spin-flip tries divided by the total number of tries in that bin, and the error bars correspond to the 68% binomial confidences. The line and confidence band (68%) are taken from a Voigt profile fit by maximum likelihood analysis of the unbinned data.

We use maximum likelihood estimation to fit the centre values of *ν*_MW_ − *ν*_*i*_(*ν*_c_∣*Γ*_*e*_, *Γ*_*I*_, *ν*_HFS_) from the three recorded resonances shown in Fig. 3 to extract *Γ*_*e*_, *Γ*_*I*_ and *ν*_HFS_, where

2 $${\varGamma }_{e}=\frac{{g}_{s}}{2}\frac{e}{q}\frac{m}{{m}_{{\rm{e}}}},\,\,{\varGamma }_{I}=\frac{{g}_{I}^{{\prime} }}{{g}_{s}}\frac{{m}_{{\rm{e}}}}{{m}_{{\rm{p}}}},$$

compare equation (1) and Supplementary Information for details. The statistical uncertainties are 17 mHz, 26 mHz and 86 mHz for *ν*_1_, *ν*_2_ and *ν*_3_, respectively. Experimental systematic shifts and uncertainties are because of special relativity effects, electrostatic and magnetostatic field imperfections, induced image charges, the axial frequency determination and the accuracy of the GPS-locked rubidium clock. Moreover, we include a second-order effect arising from the electric quadrupole moment of the nucleus^29^, slightly shifting the transition frequencies. A discussion of systematic effects and the error budget is provided in the Supplementary Information. The corrected results are *Γ*_*e*_ = −5479.8633435(11)(19), *Γ*_*I*_ = 2.1354753854(11)(3) × 10^−4^ and *ν*_HFS_ = − 12796971342.630(50)(15) Hz, where the first number in parentheses is the statistical error and the second is the systematic uncertainty.

Both the *g*-factor of the free electron and of the bare nucleus need to be corrected for the influence of the binding potential in the composite system^30^. Our calculations of the bound-electron *g*-factor include corrections due to special relativity, QED, nuclear recoil and structure effects (Supplementary Information). We evaluate *g*_*s*_(^9^Be^3+^) = −2.0017515747, where uncertainties due to uncalculated higher-order QED corrections as well as nuclear corrections are smaller than 1 in the last given digit. The binding corrections to the bare nuclear *g*-factor *g*_*I*_ are expressed as *g*′_*I*_ = (1 − *σ*)*g*_*I*_, where *σ* is the diamagnetic shielding parameter. The theoretical calculations of diamagnetic shielding parameters include corrections due to nuclear recoil, relativistic effects, one-loop QED and finite nuclear size effects. We evaluate *σ*(^9^Be^3+^) = 71.15397(14) × 10^−6^ (Supplementary Information).

From *Γ*_*I*_, we calculate the bare nuclear *g*-factor using the proton-to-electron mass ratio^31^, our values of the bound-electron *g*-factor *g*_*s*_(^9^Be^3+^) and the shielding *σ*(^9^Be^3+^). Our result, *g*_*I*_ = −0.78495442296(42)_exp_(11)_theo_, improves the accuracy by a factor of 45 compared with the result in ref. ^22^, the latter being limited by the shielding parameter of ^9^Be^+^. With a fractional uncertainty of 0.6 ppb, our result establishes the nuclear magnetic moment of ^9^Be as the second most precise, to our knowledge, surpassed only by that of the proton^32^. By comparisons with ^9^Be^+^, we evaluate the shielding factor of the lithium-like system,

3 $$1-\sigma ({}^{9}{\rm{B}}{{\rm{e}}}^{+})=(1-\sigma ({}^{9}{\rm{B}}{{\rm{e}}}^{3+}))\frac{{\varGamma }_{I}({}^{9}{\rm{B}}{{\rm{e}}}^{+})}{{\varGamma }_{I}({}^{9}{\rm{B}}{{\rm{e}}}^{3+})}\frac{{g}_{s}({}^{9}{\rm{B}}{{\rm{e}}}^{+})}{{g}_{s}({}^{9}{\rm{B}}{{\rm{e}}}^{3+})}.$$

This requires the experimental result of *Γ*_*I*_(^9^Be^+^) from ref. ^5^ and the bound-electron *g*-factor *g*_*s*_(^9^Be^+^) = −2.0022621287(24). For the latter, we use the calculations performed in ref. ^22^ and the updated nuclear recoil correction^33,34^. We evaluate *σ*(^9^Be^+^) = 141.8821(11)_exp_(12)_theo_ × 10^−6^, where the second uncertainty is limited by *g*_*s*_(^9^Be^+^). The theoretical value, *σ*(^9^Be^+^) = 141.85(3) × 10^−6^ (ref. ^22^), is in good agreement with our experimental result. To our knowledge, this constitutes the first high-precision test of the calculation of a multi-electron diamagnetic shielding parameter. The shielding calculations for the three-electron systems ^9^Be^+^ and ^6,7^Li are performed identically and use explicit values of the leading- and lowest-order recoil terms and an estimate of the relativistic correction^22,33^. At the current state of theoretical calculations of the lithium-like shielding parameters, we confirm the leading-order calculation and the estimate of the relativistic correction, solidifying its use for ^6,7^Li. In the future, the advanced calculations performed for ^3^He can be extended to ^9^Be^+^ (ref. ^35^), and our experimental value of *σ*(^9^Be^+^) will serve as an ideal benchmark at the ppb precision level.

Calculations of the zero-field hyperfine splitting can be expressed as^36–38^

4 $${\nu }_{{\rm{HFS}}}=\frac{{E}_{{\rm{F}}}}{2h}(1+{\delta }_{{\rm{pt}}}-2Z\,{\mathop{r}\limits^{ \sim }}_{Z}/{a}_{0}),$$

where *E*_F_ is the non-relativistic value of the hyperfine splitting^39^, *a*_0_ is the Bohr radius, *δ*_pt_ summarizes all corrections with a point-like treatment of the nucleus (Supplementary Information), and all nuclear structure contributions are absorbed in $-2Z{\widetilde{r}}_{Z}/{a}_{0}\approx 6\times 1{0}^{-4}$ by the effective Zemach radius ${\widetilde{r}}_{Z}$. In comparison, the relative nuclear structure contributions to the electron *g*-factor *g*_*s*_ and the shielding *σ*(^9^Be^3+^) are below 10^−10^, which highlights the sensitivity of the HFS to effects from the nuclear structure. From our experimental result, *ν*_HFS_(^9^Be^3+^) = −12796.971342630(52) MHz, we calculate the effective Zemach radius ${\widetilde{r}}_{Z}=4.048(2)\,{\rm{fm}}$. This value is consistent with that extracted from ^9^Be^+^, ${\widetilde{r}}_{Z}=4.03(5)\,{\rm{fm}}$ (ref. ^36^) (value corrected with our more accurate magnetic moment) and improves its accuracy by a factor of 25, which is possible because of the more accurate calculation of *δ*_pt_(^9^Be^3+^) compared with *δ*_pt_(^9^Be^+^).

Following investigations on ^209^Bi (ref. ^19^), we form a specific difference between the hydrogen- and lithium-like systems, Δ*ν*_HFS_ = *ν*_HFS_(^9^Be^+^) − *ξν*_HFS_(^9^Be^3+^) to cancel the large theoretical uncertainties due to nuclear structure with the calculated weighting factor *ξ* = 0.04881891046 (Supplementary Information). This complements the high-*Z* case of ^209^Bi because of the different theoretical approaches used to calculate the lithium-like HFS. For high *Z*, the large relativistic effects are included directly in the leading order by the use of a relativistic wave function basis set, whereas electron–electron correlations are treated perturbatively^40^. By contrast, for low *Z*, the electron–electron correlations lead to considerably larger contributions that require using wave functions constructed from an explicitly correlated, non-relativistic basis set, and the relativistic corrections are instead treated perturbatively^41,42^. Therefore, in the case of ^209^Bi, higher-order QED terms are tested by Δ*ν*_HFS_, whereas in our study at low *Z*, higher-order electron correlation effects are benchmarked. The experimental result, using *ν*_HFS_(^9^Be^+^) = −625.008837044(12) MHz from ref. ^43^ is Δ*ν*_HFS,exp_ = −274.638909(12) kHz, where the uncertainty is dominated by *ν*_HFS_(^9^Be^+^). We calculate the theoretical value, Δ*ν*_HFS,theo_ = −271.4(3.6) kHz, which is in good agreement with the experimental value but has a much larger uncertainty. Equivalently, this constitutes a test of *ν*_HFS_(^9^Be^+^) with 6-ppm precision. Similar to calculations for ^6,7^Li, estimates of the QED contributions to the lithium-like system limit the accuracy of the theoretical result^38^.

**Fig. 4:**
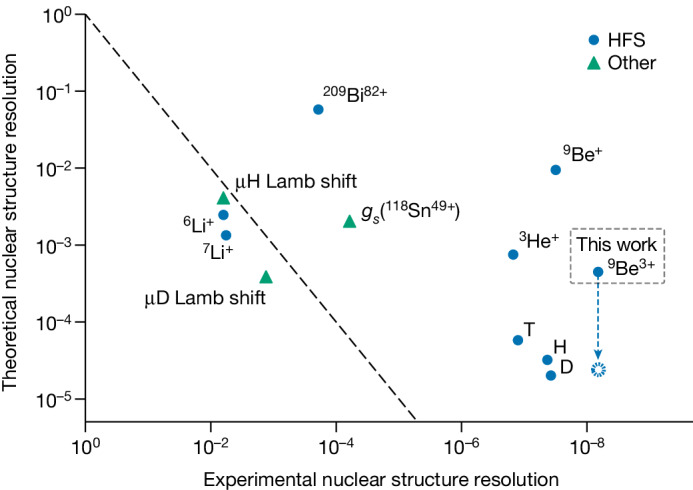
Theoretical and experimental nuclear structure resolution of various measurements^1–4,7,8,14,19,36–38,43,49,50^. The *y*-axis is the fraction of the theoretical uncertainty of the point-nucleus calculations to the nuclear structure contribution. The *x*-axis is the fraction of experimental uncertainty to the nuclear structure contribution. The dashed diagonal line indicates equal experimental and theoretical resolution. For H, D and T, HFS calculations from ref. ^38^ were used, which, as opposed to our value for *ν*_HFS_(^9^Be^3+^), do not include contributions and uncertainties from hadronic and muonic vacuum polarization as well as certain nuclear recoil terms. For comparison, the blue dashed line and point indicate the reduced theoretical uncertainty of *ν*_HFS_(^9^Be^3+^) using the same calculations that do not include the aforementioned contributions. For details, see Supplementary Table 7.

In Fig. 4, we compare the nuclear structure resolution of several measurements. Our determination of the nuclear structure contribution to the HFS of ^9^Be by ^9^Be^3+^ shows leading experimental resolution and substantial improvements of the theory compared with ^9^Be^+^. This progress now enables testing other HFS systems in ^9^Be with higher accuracy using our precise values of the Zemach radius and the nuclear magnetic moment, as demonstrated by our evaluation of the specific difference. Furthermore, future tests of the theory of the HFS in the helium-like system ^9^Be^2+^ will benefit from our results^44^, and direct measurements of the 2*s* HFS in ^9^Be^3+^ would provide an opportunity for highly stringent tests of bound-state QED because of the more accurate theory in single-electron systems.

Finally, we calculate the atomic mass of ^9^Be from *Γ*_*e*_, using the electron mass^31^, the required binding energies^45–47^ and our theoretical value of *g*_*s*_(^9^Be^3+^). The result, ${m}_{{}^{9}{\rm{Be}}}=9.0121830344(35)\,{\rm{u}}$, is in perfect agreement with the accepted value^23^ and improves the uncertainty by a factor of 20.

The results are summarized in Table 1. In conclusion, our precision measurement of the bare nuclear magnetic moment and the effective Zemach radius of ^9^Be with hydrogen-like ^9^Be^3+^ enables tests of QED methods accessible only with accurate knowledge of these properties. At present, by comparison with measurements on ^9^Be^+^, we provide the, to our knowledge, first high-precision test of multi-electron diamagnetic shielding calculations and a 6-ppm test of the QED calculations of the lithium-like 2*s* HFS. High-precision Penning-trap measurements of hyperfine and Zeeman splittings, which we previously demonstrated for ^3^He^+^, are now possible for a multitude of other hydrogen-like or lithium-like ions, enabling the suppression of nuclear effects, mandatory for further applications such as spectroscopic searches for physics beyond the standard model^48^. Furthermore, measurements on ^6,7^Li^2+^ would enable direct comparisons with the recent measurements on the helium-like charge states^21,37,49^.

**Table 1:** Summary of results

Value	References
${g}_{I}=-0.78495442296{(42)}_{\exp }{(11)}_{{\rm{theo}}}$	TW
*g*_*I*_ = −0.78495439(2)_theo_	Ref. ^22^
$\sigma ({}^{9}{\rm{B}}{{\rm{e}}}^{+})=141.8821(11{)}_{\exp }(12{)}_{{\rm{theo}}}\times {10}^{-6}$	TW, refs. ^5,22,34^
*σ*(^9^Be^+^) = 141.85(3)_theo_ × 10^−6^	Ref. ^22^
${\widetilde{r}}_{Z}=4.048(2)\,{\rm{fm}}$	TW
${\widetilde{r}}_{Z}=4.03(5)\,{\rm{fm}}$	Ref. ^36^
$\Delta {\nu }_{{\rm{HFS,exp}}}=-274.638909(12)\,{\rm{kHz}}$	TW, ref. ^43^
Δ*ν*_HFS,theo_ = −271.4(3.6) kHz	TW, ref. ^36^
${m}_{{}^{9}{\rm{Be}}}=9.0121830344(35)\,{\rm{u}}$	TW
${m}_{{}^{9}{\rm{Be}}}=9.01218306(8)\,{\rm{u}}$	Ref. ^23^

TW refers to results derived in this work.

## Online content

Any methods, additional references, Nature Portfolio reporting summaries, source data, extended data, supplementary information, acknowledgements, peer review information; details of author contributions and competing interests; and statements of data and code availability are available at 10.1038/s41586-024-07795-1.

## Supplementary information

Supplementary Information

## Data Availability

The datasets generated and analysed during this study are available from the corresponding author upon request.

## Code availability

The code used during this study is available from the corresponding author upon reasonable request.
