# Publisher Correction: Precision spectroscopy on ^9^Be overcomes limitations from nuclear structure

**DOI:** 10.1038/s41586-024-08018-3

**Published:** 2024-09-10

**Authors:** Stefan Dickopf, Bastian Sikora, Annabelle Kaiser, Marius Müller, Stefan Ulmer, Vladimir A. Yerokhin, Zoltán Harman, Christoph H. Keitel, Andreas Mooser, Klaus Blaum

**Affiliations:** 1 Max Planck Institute for Nuclear Physics, Heidelberg, Germany; 2 Institute for Experimental Physics, Heinrich Heine University Düsseldorf, Düsseldorf, Germany; 3 Ulmer Fundamental Symmetries Laboratory, RIKEN, Saitama, Japan

**Keywords:** Atomic and molecular physics, Quantum physics

Correction to: *Nature* 10.1038/s41586-024-07795-1 Published online 14 August 2024

In the version of the article originally published, in the text now reading “The corrected results are *Γ*_*e*_ = −5479.8633435(11)(19), *Γ*_*I*_ = 2.1354753854(11)(3) × 10^−4^ and *ν*_HFS_ = −12796971342.630(50)(15) Hz,” *Γ*_*e*_  and *Γ*_*I*_ were listed with units (Hz) where no units were required. The text has been amended in the HTML and PDF versions of the article.

