# Supplementary material for: Precision spectroscopy on 9Be overcomes limitations from nuclear structure
Source: Nature. 2024 Aug 14;632(8026):757–61. doi: 10.1038/s41586-024-07795-1 (PMC11338825; doi:10.1038/s41586-024-07795-1)
Supplement: Supplementary file 1 — Supplementary Information [file 41586_2024_7795_MOESM1_ESM.pdf]

nature portfolio

https://doi.org/10.1038/s41586-024-07795-1

Supplementary information

Precision spectroscopy on ^{9}Be overcomes limitations from nuclear structure

In the format provided by the
authors and unedited

1 Supplement - Experiment

Resonance fitting and statistical uncertainties

In each measurement cycle, we measure the cyclotron frequency ν_c while irradiating microwaves at frequency ν_{MW} and subsequently determine whether a change of spin state occurred. From the former two values we form the quantity $\tilde{\Delta}_i = \nu_{\mathrm{MW}} - \nu_i(\nu_c, \tilde{\vec{p}})$, with a suitable guess $\tilde{\vec{p}} = (\tilde{\Gamma}_e, \tilde{\Gamma}_I, \tilde{\nu}_{\mathrm{HFS}})$ close to the real parameters $\vec{p} = (\Gamma_e, \Gamma_I, \nu_{\mathrm{HFS}})$. The probability to observe a spin-state change is described by the Rabi cycle

$$P(\Delta_i, t) = \frac{\Omega^2}{\Omega^2 + \Delta_i^2} \sin^2\left(\pi\sqrt{\Omega^2 + \Delta_i^2}t\right), \tag{S1}$$

where $\Delta_i = \nu_{\mathrm{MW}} - \nu_i(\nu_c, \vec{p})$, Ω is the Rabi frequency and t the microwave irradiation time. The uncertainty of the magnetic field measurement through ν_c leads to a gaussian distributed Δ_i with width $\sigma(\Delta_i) \approx \frac{\partial\nu_i(\nu_c)}{\partial\nu_c}\sigma(\nu_c)$, which is added to the above probability via convolution. In the limit of $\Omega \lessapprox \sigma(\Delta_i)$ and $t > 1/\Omega$ this leads to a Voigt profile with Gaussian width $\sigma(\Delta_i)$ and Lorentzian width Ω centered at $\Delta_i = 0$ for a fixed time t. The maximum probability converges to 0.5 for large times, but as can be seen from Fig. 3, this is not necessarily the case here. To check our understanding of the probability lineshape we explicitly measure the Rabi cycle for the ν_1 transition at a Rabi frequency $\Omega \approx 0.44$ Hz $> \sigma(\Delta_i) \approx 0.065$ Hz, see Figure S1. With the smaller Rabi frequencies used for the

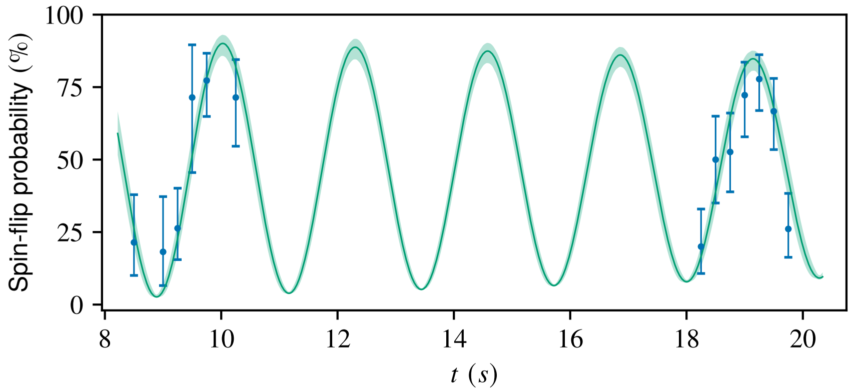

Figure S1: Measured Rabi cycle of the ν_1 transition. The fit is done with the probability (S1) convolved over Δ with a Gaussian. The shaded region shows the 1-σ confidence band of the fit. We observe good agreement with our model.

resonances in Fig. 3, a weak time dependence, which was not explicitly optimized, on the reached amplitude is still expected for all transitions.

We use a maximum likelihood estimation (MLE) and a complementary Markov chain Monte Carlo method, see ref. [1], to fit the center $\langle\tilde{\Delta}_i\rangle$, $i = \{1, 2, 3\}$ together with the widths and amplitudes of the individual resonances with a Voigt profile. Both methods produce identical results on the level of the significant digits of the statistical result. To simultaneously fit the most likely values $\vec{p}$, we adjust $\tilde{\vec{p}}$ such that the fitted center values $\langle\tilde{\Delta}_i(\tilde{\vec{p}})\rangle = \langle\Delta_i(\vec{p})\rangle = 0$.

The statistical covariance matrix of the parameters is estimated via the non-linear least square estimator to be

$$\mathrm{cov}(\vec{p})_{\mathrm{stat}} = J^{-1}\mathrm{cov}(\vec{\Delta})_{\mathrm{stat}}{J^{-1}}^T, \tag{S2}$$

where

$$J_{ij}(\vec{p}) = \frac{\partial\nu_i}{\partial p_j}(\vec{p}) \tag{S3}$$

is the Jacobi matrix and $\mathrm{cov}(\vec{\Delta})_{\mathrm{stat}} = \mathrm{diag}(\sigma^2(\Delta_i))$ is the diagonal matrix of the variances of the fitted centers.

Systematic shifts

We calculate the Zeeman and hyperfine transition frequencies via the measured cyclotron frequency ν_c. As the transition frequencies depend on the magnetic field at the position of the ion $B_{\rm ion}$, we need to consider systematic shifts of the form $\nu_c = \frac{1}{2\pi}\frac{q}{m}B_{\rm ion} + \delta\nu_c$. The individual shifts of the motional frequencies are propagated by the invariance theorem $\nu_c^2 = \nu_+^2 + \nu_-^2 + \nu_z^2$ to the free cyclotron frequency. Shifts of the free cyclotron frequency $\delta\nu_c$ then shift our fitted center by

$$\langle\Delta_i\rangle \rightarrow \langle\Delta_i\rangle - \frac{\partial\nu_i}{\partial\nu_c}\delta\nu_c = \langle\Delta_i\rangle + \delta\nu_i. \tag{S4}$$

Similar to the treatment of the statistical fitting we use the Jacobian matrix, Eq. (S3), to compute the shift on the parameters as

$$\vec{\delta p} = J^{-1}\vec{\delta\nu}. \tag{S5}$$

For the uncertainties we propagate the full covariances to the parameters, similar to the uncertainty of the statistical result, Eq. (S2),

$$\mathrm{cov}(\vec{p})_{\rm syst} = J^{-1}\mathrm{cov}(\vec{\delta\nu})_{\rm syst}{J^{-1}}^T. \tag{S6}$$

Here $\mathrm{cov}(\vec{\delta\nu})_{\rm syst}$ is the covariance matrix of the systematic shifts on the resonance centers. This is necessary, as we have correlated uncertainties, which include the image charge shift and uncertainties of the inhomogeneites and anharmonicity values, but also uncorrelated uncertainties from shifts depending on the thermal radii of the ion and the uncertainty of the time standard. In particular, the shifts of ν_c lead to full cancellation in the value of $\nu_{\rm HFS}$ and Γ_I, see Table S1. Only the uncorrelated uncertainties of ν_c translate to uncertainties in Γ_I and $\nu_{\rm HFS}$.

For shifts due to magnetic field imperfections, only the shift of ν_z by B_2 due to the finite amplitude of the modified cyclotron, ρ_+, and magnetron mode, ρ_-,

$$\frac{\delta\nu_z}{\nu_z} = \frac{B_2}{4B_0}\left(\frac{\nu_c}{\nu_-}\rho_+^2 + \frac{\nu_c}{\nu_p}\rho_-^2\right), \tag{S7}$$

is relevant [2]. Shifts of the motional frequencies due to non-harmonic contributions in the electrostatic potential $\Phi(z) = C_2z^2 + \sum_{k>2}C_kz^k$ need to be considered as well. The lowest order shifts are

$$\begin{aligned}\delta\nu_+ &= \frac{3}{2}\frac{C_4}{C_2}\nu_-\left(-2\rho_z^2 + \rho_+^2 + 2\rho_-^2\right),\\ \delta\nu_z &= \frac{3}{4}\frac{C_4}{C_2}\nu_z\left(\rho_z^2 - 2\rho_+^2 - 2\rho_-^2\right) + \frac{1}{16}\frac{C_3^2}{C_2^2}\nu_z\left(-15\rho_z^2 + 18\rho_+^2 + 18\rho_-^2\right),\end{aligned} \tag{S8}$$

where ρ_z is the amplitude of the axial oscillation [2]. We use a 7-pole compensated Penning trap which has two pairs of correction electrodes to cancel the leading anharmonicities, see ref. [3] for a similar design. To this end we excite the radius of the magnetron motion ρ_- and measure the dependencies $\nu_z(\rho_-^2, \rho_-^4)$ which is optimized to zero by adjusting the two voltages applied to the respective correction electrode pairs, see e.g. S2. Similarly, by measuring the dependence $\nu_z(\rho_z^2)$ for different correction electrode voltages, we can differentiate between the C_3 and C_4 terms in Eq. (S8). The value of $B_2 \approx 1\ \mathrm{T/m^2}$ is measured by comparing its influence on ν_p and ν_z. All frequency shifts due to higher order field imperfections can be neglected at the current experimental precision.

The free cyclotron frequency ν_c is shifted due to the relativistic mass increase to smaller observed frequencies [2]

$$\nu_c' = \frac{1}{\gamma}\nu_c \approx \left(1 - \frac{1}{2}\frac{(2\pi\nu_c)^2\rho_+^2}{c^2}\right)\nu_c, \tag{S9}$$

where c is the speed of light in vacuum and γ is the Lorentz factor. Typically we have small shifts $\gamma - 1 < 10^{-11}$. A hyperfine transition is on resonance, if the microwave frequency $\nu_{\rm MW}'$ in the ions rotating (rest) frame is equal to the transition frequency $\nu_i(B')$ at the ion's position. In the rotating frame the magnetic field is perceived as the boosted quantity $B' = \gamma B$ and the microwave frequency

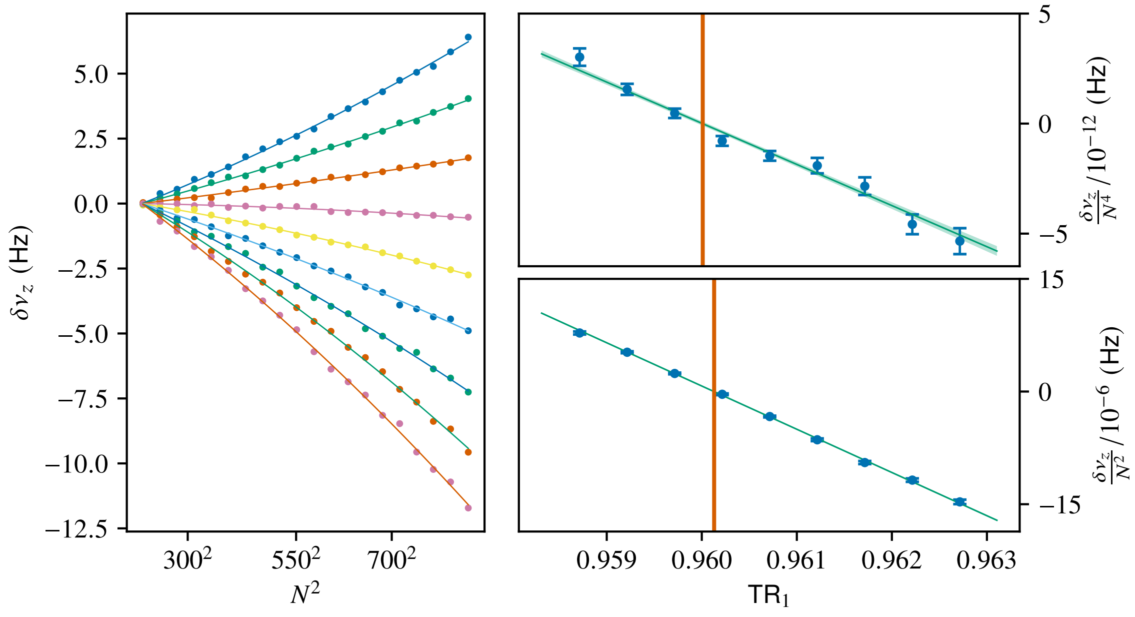

Figure S2: Optimization of the correction electrode voltages. Here, the tuning ratios $\mathrm{TR}_{1,2}$ are defined as the ratio between the voltage on the correction electrode pair to the voltage on the central (ring) electrode. On the left, $\delta(\nu_z)(N^2)$, where $\rho_- \propto N$, is measured for different values of TR_1 and a polynomial fit is used to determine the dependencies on ρ_-^2 and ρ_-^4. On the right, these dependencies are plotted against TR_1 to determine the intercepts (colored in red), where no shifts occur. The intercept of $\delta(\nu_z)/N^2 = 0$ is used as the optimum to give $C_4 = 0$. The small difference to the $\delta(\nu_z)/N^4 = 0$ intercept results in a negligible residual higher order anharmonicity C_6.

is shifted by the transverse Doppler effect to $\nu'_{\mathrm{MW}} = \gamma\nu_{\mathrm{MW}}$. If we use the observed (not corrected for relativistic shift) ν'_c for our fit of $\langle\Delta'_i\rangle$, the first order relativistic shift evaluates to a shift of the center value

$$\langle\Delta'_i\rangle = \langle\Delta_i\rangle - (\gamma - 1)\left(\nu_i - 2\nu_c\frac{\partial\nu_i}{\partial\nu_c}\right). \tag{S10}$$

Higher order effects, in particular the Thomas precession which contributes about 3×10^{-12} to Γ_I, can be neglected at our current precision.

The above shifts depend on the thermal radii of the ion, which are related to the temperature of the detection system at ~ 4.2 K. In practice, the related temperature can be quite a bit higher, owing to e.g. heating from cryogenic amplifiers. We determine the temperature of the axial detection system in the PT by measuring the energy distribution of the cyclotron mode in the AT. During each cycle of our measurement we have to compensate for the strong $B_{2,\mathrm{AT}} \approx 282\ \mathrm{kTm}^{-2}$ induced frequency shift, Eq. (S7), by adjusting the ring voltage to bring the ion into resonance with the AT detection system. From an MLE fit to the distribution of ring voltages, see S3, we determine the temperature. We use the mean temperature value determined from the three resonances and assign a conservative uncertainty of the largest difference between the measurements. The value $T_z = 6.28(30)$ K corresponds to the thermal amplitude $\rho_z = 35.41(85)$ µm which is related to the modified cyclotron and magnetron amplitudes via $\rho_\pm = \sqrt{\nu_z/\nu_+}\rho_z$.

A shift of the motional frequencies arises from to the image charges induced on the trap surfaces due to the Coulomb force of the ion. We calculate the relative shift on the cyclotron frequency to be $\delta\nu_c/\nu_c = 95.8(4.8) \times 10^{-12}$, where the uncertainty of this shift is typically taken to be 5% [4].

The axial and radial frequencies are determined by fits of a thermal dip lineshape to the Fourier spectrum of our detection signal [5]. This lineshape can be modelled with additional parameters, which account for e.g. amplification transfer functions, non-ideal noise short circuits by the ion, etc. We estimate an upper bound for this systematic uncertainty on the cyclotron frequency to be $\sigma(\nu_c) = 10$ mHz.

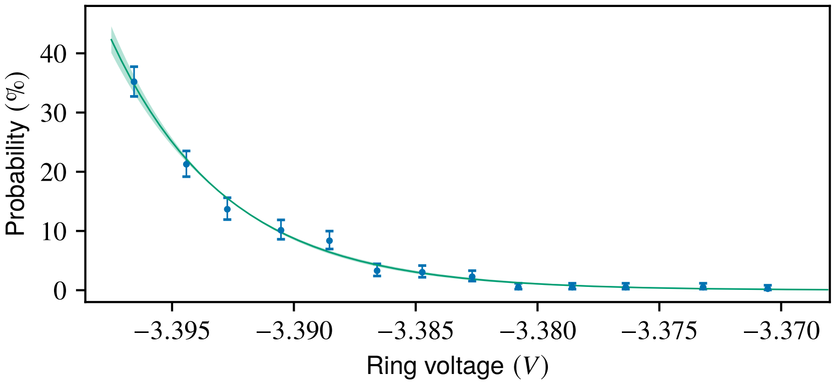

Figure S3: Measured distribution of voltages in the AT. The fit is performed via MLE. For details see text.

Our detection system and all signal generators are connected to a rubidium atomic clock, which in turn is locked to a GPS reference clock. Any relative frequency offset δ_{ref} of the clock leads to the following shift

$$\Delta_i' \approx \Delta_i + \delta_{\mathrm{ref}}(\nu - \nu_c \frac{\partial \nu_i}{\partial \nu_c}) \tag{S11}$$

The GPS locked rubidium clock, model FS725 from SRS, is rated at 10^{-12} accuracy and stability over days of measurement time, so a correlated and uncorrelated uncertainty $\sigma(\delta_{\mathrm{ref}})_{\mathrm{corr}} = \sigma(\delta_{\mathrm{ref}})_{\mathrm{uncorr}} = 10^{-12}$ among the three measured center values may exist and has to be taken into account. While a common/correlated uncertainty of the reference only leads to an uncertainty of ν_{HFS}, the uncorrelated uncertainty gives rise to shifts on all parameters, and especially, leads to large uncertainty for Γ_I.

In ref. [6], second-order corrections to the energy levels of the Zeeman and hyperfine splitting are discussed. Including the non-zero nuclear electric quadrupole moment $Q(^9\mathrm{Be}) = 0.0529(4) \times 10^{-28}\mathrm{m}^{-2}$ [7] leads to small shifts of the transition frequencies. Comparing the frequencies as calculated from ref. [6], $\nu_{Q,i}$, with the classical Breit-Rabi derivation gives, $\nu_{Q,1} - \nu_1 = -36.4(3)$ mHz, $\nu_{Q,2} - \nu_2 = 5.69(4)$ mHz and $\nu_{Q,3} - \nu_3 = 4.36(3)$ mHz.

Table S1 lists the statistical results and the systematic shifts. Additionally, we summarize the

Table S1: Statistical results and error budget. For more details, see Supplementary Information.

	$\boldsymbol{\Gamma_e}$	$\boldsymbol{\Gamma_I}$	$\boldsymbol{\nu_{\mathrm{HFS}}}$
**Statistical result**	$-5479.8633446(11)$	$2.1354753839(11) \times 10^{-4}$	$-12796971342.569(50)$ Hz
**Systematic shifts**	$/10^{-7}$	$/10^{-14}$	/mHz
Field imperfections	$-1(<1)$	$0(<1)$	$0(<1)$
Relativistic	$0(<1)$	$0(1)$	$50(3)$
Image charge	$-5(<1)$	-	-
Dip	$0(19)$	-	-
Frequency reference	$0(1)$	$0(3)$	$0(15)$
Quadrupole moment	$-5(<1)$	$-15(<1)$	$11(<1)$
Total shifts	$-11(19)$	$-15(3)$	$61(15)$
**Corrected result**	$-5479.8633435(22)$	$2.1354753854(11) \times 10^{-4}$	$-12796971342.630(52)$ Hz

correlation via the Pearson correlation coefficients $\rho(X,Y) = \frac{\mathrm{cov}(X,Y)}{\sigma(X)\sigma(Y)}$ [8], see Table S2.

Table S2: Correlation coefficients of the fit parameters.

	statistical	systematic	combined
$\rho(\Gamma_e, \Gamma_I)$	0.48	0.04	0.24
$\rho(\Gamma_e, \nu_{\mathrm{HFS}})$	0.80	-	0.38
$\rho(\Gamma_I, \nu_{\mathrm{HFS}})$	0.60	0.14	0.56

2 Supplement theory

Hyperfine splitting and effective Zemach radius

The energy difference between the two ground-state hyperfine sublevels of the $^9\mathrm{Be}^{3+}$ ion at zero magnetic field can be parametrized by

$$E_{\mathrm{HFS,theo}} = 2h\nu_{\mathrm{HFS}} = E_{\mathrm{F}}(A(Z\alpha) + \delta_{\mathrm{recoil,pt}} + \delta_{\mathrm{QED,pt}} + \delta_{\mu\mathrm{VP}} + \delta_{\mathrm{had\,VP}} + \delta_{\mathrm{FS}})\,. \tag{S12}$$

For the $1s$ state and a nuclear spin of $I = 3/2$ [9], the non-relativistic Fermi energy E_{F} is given by [10]

$$E_{\mathrm{F}} = \alpha g_I \frac{m_e}{m_p}\frac{8}{3} m_e c^2 (Z\alpha)^3 \mathcal{M}\,. \tag{S13}$$

Here, α is the fine-structure constant.

The relativistic factor can be calculated analytically, using wave functions for the model of a point-like nucleus. For the $1s$ state, it is [10]

$$A(Z\alpha) = \frac{1}{\gamma(2\gamma - 1)}\,, \tag{S14}$$

with $\gamma = \sqrt{1-(Z\alpha)^2}$.

The finite nuclear mass (recoil) correction is partially taken into account by the mass factor $\mathcal{M} = (1 + m_e/M_N)^{-3}$ [10], with the nuclear mass M_N. For recoil corrections beyond the mass factor and to leading order in $Z\alpha$, we use the formula developed for systems with point-like nuclei (e.g. muonium) [11]. Higher-order (in $Z\alpha$) recoil corrections are treated as in refs. [12–14]. Since, to our knowledge, radiative recoil corrections to HFS have not been calculated for nuclei with spin different from 1/2, we used the point-nucleus formula for spin-1/2 from ref. [11] as the uncertainty due to uncalculated radiative recoil corrections.

The QED corrections can be parametrized as [15]

$$\delta_{\mathrm{QED}} = a_e + \delta_{\mathrm{QED,binding}}\,, \tag{S15}$$

with the free electron anomaly a_e [16]. One-loop binding corrections were calculated to all orders in $Z\alpha$. Specifically, the one-loop self-energy was calculated as the sum of the perturbative $Z\alpha$ expansion formula as given in refs. [10, 17] and the tabulated higher-order term from ref. [17]. In ref. [18], we calculated the vacuum polarization (VP) correction for the model of a point-like nucleus, with the VP loop in the Uehling approximation [19]. The electric-loop contribution was calculated using the electron wave function perturbed by the nuclear magnetic field, as given in ref. [20, 21]. The magnetic loop correction was calculated following refs. [10, 22]. We found our VP results for various nuclear charge numbers to be in excellent agreement with results from ref. [10, 21]. The above SE and VP results were obtained for the point-like nuclear model.

Two-loop binding corrections have been calculated up to order $(Z\alpha)^2$ [15, 23, 24]. Apart from the (for the purpose of our calculation negligibly small) uncertainty of a_e [16], we took into account the uncertainty of the one-loop self-energy correction as given in ref. [17] and the given uncertainties of the two-loop $Z\alpha$ expansion coefficients [15, 24]. We estimated the uncertainty of the QED parameter due to uncalculated two-loop binding corrections of orders $(Z\alpha)^3$ and higher as $2\,(\alpha/\pi)^2\,(Z\alpha)^3 \log\left[(Z\alpha)^{-2}\right] \approx 1.9 \times 10^{-9}$. In a similar way, the uncertainty due to uncalculated 3-loop binding corrections was

Table S3: Contributions to the ground-state hyperfine splitting in $^9Be^{3+}$. All contributions listed here assume a point-like nucleus.

Contribution			Following refs.
Fermi energy	$E_F/h =$	-25 558 815.5 kHz	[16]
Relativistic	$A(Z\alpha) - 1 =$	0.001 279 577	[10, 25]
QED	$\delta_{QED} =$	0.000 722 219(12)	[10, 15–17, 26]
Recoil	$\delta_{recoil} =$	-0.000 017 38(24)	[11–13]
Muonic VP	$\delta_{\mu VP} =$	0.000 000 78(8)	[18]
Hadronic VP	$\delta_{had VP} =$	0.000 001 11(11)	[18]

estimated as $2\left(\alpha/\pi\right)^3(Z\alpha) \approx 7.3 \times 10^{-10}$. In total, we obtain the QED correction parameter $\delta_{QED} = 0.000\,722\,219(12)$.

The parameters for the muonic and hadronic vacuum polarization corrections $\delta_{\mu VP}$ and $\delta_{had VP}$ in Table S3 correspond to the sum of the muonic/hadronic Uehling potential correction and the leading-order magnetic loop correction for the point-like nuclear model and are taken from ref. [18]. We include both parameters with a relative uncertainty of 10% to account for uncalculated light-by-light scattering type muonic and hadronic vacuum polarization contributions.

All contributions discussed above assume a point-like treatment of the nucleus and are summarized in Table S3. We incorporate all corrections to the HFS related to the finite size of the nucleus (nuclear structure) into the effective Zemach radius $\tilde{r}_Z$, defined following ref. [27] as,

$$\delta_{FS} = -2Z\tilde{r}_Z/a_0\,. \tag{S16}$$

Apart from the finite size of the nuclear charge distribution, this includes the dominant Bohr-Weisskopf effect, nuclear structure dependent recoil effects, and additional QED finite-size cross-terms as well as nuclear polarization corrections. From the experimental value of the hyperfine splitting in our work, assuming $E_{HFS,theo} = E_{HFS,exp}$, we find the effective Zemach radius to be $\tilde{r}_Z = 4.048(2)$ fm. The uncertainty is dominated by uncalculated radiative recoil contributions (see discussion above).

Weighted difference of the hyperfine splittings

For ^{9}Be, with a new value for the HFS in the hydrogen-like charge state in our work, and a high-precision measurement of the HFS in the lithium-like charge state in ref. [28], we put forward a weighted difference of hyperfine splittings in such a way that nuclear effects are strongly suppressed, see ref. [29], and compare the experimental value for the weighted difference with the theoretical value for the weighted difference.

The theoretical value for the hyperfine splitting of the Li-like Be ion can be parametrized following ref. [27]

$$\begin{aligned} E_{HFS,2s} =& E_{F,2s}(A_{2s}(Z\alpha) - 2\alpha Z\tilde{r}_Z + \delta_{recoil,pt} + \delta_{QED,2s,pt}) = \\ & \frac{m_e^2 g_I}{Z m_p}\left(\frac{g_e}{2}\alpha^4 A^{(4)} + \sum_{n=5}^{\infty}\alpha^n A^{(n)}\right)\,, \end{aligned} \tag{S17}$$

with the free electron g-factor $g_e = 2(1 + a_e)$. The first parametrization was chosen to resemble our parametrization of the HFS in the hydrogen-like charge state from the previous section. The second parametrization is taken from ref. [27]. In that work, explicit values are given for the coefficients $A^{(4)}$ to $A^{(7)}$, with $A^{(7)}$ being an estimate based on one-electron bound QED. We first discuss how coefficients from both parametrizations are related to each other.

The leading-order contribution in the non-relativistic limit is given by

$$E_{F,2s} = \frac{m_e^2 g_I}{Z m_p}\alpha^4 A^{(4)}\,, \tag{S18}$$

with the numerical value for $A^{(4)}$ tabulated in ref. [27]. Relativistic corrections to the leading order are parametrized as

$$A_{2s}(Z\alpha) = 1 + \alpha^2 \frac{A^{(6)}_{\rm rel}}{A^{(4)}}\,. \tag{S19}$$

Here, $A^{(6)}_{\rm rel}$ corresponds to a part of the total term $A^{(6)}$ which contains relativistic and QED terms as follows,

$$\begin{aligned} A^{(6)} &= A^{(6)}_{\rm rel} + A^{(6)}_{\rm R}\,, \\ A^{(6)}_{\rm rel} &= A^{(6)}_{\rm AN} + A^{(6)}_{\rm B} + A^{(6)}_{\rm C}\,. \end{aligned} \tag{S20}$$

In ref. [27], numerical values for the relativistic corrections $A^{(6)}_{\rm AN}$, $A^{(6)}_{\rm B}$ and $A^{(6)}_{\rm C}$ as well as the QED term $A^{(6)}_{\rm R}$ are tabulated. The term $A^{(6)}_{\rm R}$ corresponds to one-loop QED corrections of $\mathcal{O}(Z\alpha)$. The term $A^{(7)}$ corresponds to the sum of one-loop QED terms of $\mathcal{O}\left((Z\alpha)^2\right)$ and two-loop QED terms of $\mathcal{O}(Z\alpha)$. With this, we find the following QED parameter

$$\delta_{\rm QED,2s,pt} = a_e + \alpha^2 \frac{A^{(6)}_{\rm R}}{A^{(4)}} + \alpha^3 \frac{A^{(7)}}{A^{(4)}}\,. \tag{S21}$$

Finally, the parameter $A^{(5)}$ corresponds to the sum of nuclear finite size and recoil corrections as follows

$$\alpha \frac{A^{(5)}}{A^{(4)}} = -2 m_e Z\alpha \tilde{r}_Z + \delta_{\rm recoil,pt}\,. \tag{S22}$$

The recoil correction parameter for point nuclei is identical for the hydrogen-like and lithium-like systems.

In order to combine the hydrogen-like and the lithium-like hyperfine splitting values in a way to efficiently cancel nuclear effects, we may use the following weighted difference

$$E^{\xi}_{\rm HFS} = E_{\rm HFS,2s} - \xi E_{\rm HFS,1s}\,, \tag{S23}$$

with the weight factor

$$\xi = \frac{E_{\rm F,2s}}{E_{\rm F}} = \frac{3A^{(4)}}{8Z^4\mathcal{M}} = 0.048\,818\,910\,46\,. \tag{S24}$$

With this weight factor, the effective Zemach radius corrections from lithium-like and hydrogen-like hyperfine splitting cancel exactly.

As a *side effect* of the choice of weight factor, the leading contributions, represented by $E_{\rm F}$ and $E_{\rm F,2s}$ in the H-like and Li-like charge state respectively, cancel exactly. With this, the weighted difference is several orders of magnitude smaller than the individual HFS values. Furthermore, leading QED corrections due to the electron's magnetic moment anomaly a_e and the one-loop QED term of order $Z\alpha$ cancel in the weighted difference. With this, the contributions that do remain in the weighted difference are relativistic corrections according to $A^{(6)}_{\rm rel}$ in the lithiumlike hyperfine splitting and its corresponding hydrogenlike term, as well as higher-order QED terms $A^{(7)}$.

The leading recoil corrections obtained for the point nucleus model cancel exactly. Moreover, recent recoil calculations [30] imply that the leading-order part of the recoil parameters in equations (S12) and (S17) is identical even for extended nucleus calculations. We therefore assume an exact cancellation of the leading recoil contribution in the weighted difference.

The uncertainty due to uncalculated higher-order recoil corrections in the weighted difference was estimated based on the higher-order contribution to the recoil correction to the specific difference D_{21} of hyperfine splittings of the $2s$ and $1s$ states, as given in ref. [24].

For the uncertainty due to uncalculated higher-order nuclear size corrections, we estimated the total nuclear size charge and magnetization distribution corrections for both $1s$ and $2s$ states using semi-analytic wavefunctions for the bound electron [31] and formulas from refs. [25, 32]. We estimate an

Order	ΔE_{1s}	ΔE_{2s}		E^{ξ}_{HFS}	
$m\alpha^4$, non-rel	-25 558 815.5	-1 247 753.5		0.0	
$m\alpha^4$, a_e	-29 639.3	-1 447.0		0.0	
$m\alpha^5$, Recoil	421.0	20.6		0.0	
$m\alpha^6$, rel	-32 665.0	-2 122.3		-527.6	
$m\alpha^6$, 1-loop QED	9836.8	480.3		0.0	
$m\alpha^7$, 1-loop QED	1211.3	44.0	(7.2)	-15.1	(7.2)
$m\alpha^7$, 2-loop QED	-9.8	-0.5		0.0	

Table S4: Contributions of different orders in α to the hyperfine splitting in H-like and Li-like ^{9}Be ions and to the weighted difference, in kHz.

uncertainty due to higher-order finite size corrections to the weighted difference as $\frac{m_e^2 g_I}{2Zm_p} A^{(4)} |\delta_{FS,2s} - \delta_{FS,1s}|$.

Considering that the lithiumlike theory is developed up to order $m\alpha^7$, in the following, we estimate uncertainties of the weighted difference that may arise due to uncalculated higher-order QED terms. Explicit calculations of the one-loop (electronic) vacuum polarization correction for the $1s$ and 2s states (the latter in the one-electron approximation) for both extended and point-like nuclei imply very similar VP-FS cross terms in both cases [18]. With this, we estimate the uncertainty of the weighted difference due to QED-FS cross terms as $2\frac{m_e^2 g_I}{2Zm_p} A^{(4)} |\delta_{\text{VP,FS},2s} - \delta_{\text{VP,FS},1s}|$.

All estimated uncertainties due to higher-order finite size or recoil effects are at least one order of magnitude smaller than the estimated uncertainty of the Li-like QED theory as given in ref. [27]. For the total theoretical value of the weighted difference, taking into account the hydrogenlike QED theory up to order $m\alpha^7$, we find

$$E^{\xi}_{\text{HFS,theo}}/h = -542.7(7.2)\,\text{kHz}\,. \tag{S25}$$

The experimental value for the weighted difference is determined by inserting the experimental HFS values for the H-like ion from this work, and for the Li-like system from ref. [28].

Diamagnetic shielding

The combined effect of the external and the nuclear magnetic fields leads to a splitting of the $1s$ state into multiple magnetic sublevels. To first order in perturbation theory, these sublevels are described with the standard Breit-Rabi formulas [33]. In ref. [6], several second-order corrections to the Breit-Rabi formulas were calculated. The second-order Zeeman shift turns out to be identical for all sublevels and therefore does not have an impact on frequencies of transitions between sublevels. We also estimated the third order Zeeman splitting by extrapolating tabulated results from ref. [34] to low Z. Even for a magnetic field of 7 T, the third-order Zeeman shift corresponds to a correction to the bound-electron's g-factor of 4×10^{-15} and is therefore negligibly small.

Apart from that, the modification of the Breit-Rabi formulas due to nuclear magnetic dipole shielding as well as the nuclear electric quadrupole shielding were calculated in ref. [6]. The magnetic dipole shielding corresponds to Feynman diagrams with two magnetic interactions, namely one external magnetic field and one nuclear magnetic field line [35]. Assuming the model of a point-like nucleus, the leading value of the shielding constant can be given analytically as [36–41].

$$\sigma^{(0)} = -\frac{4\alpha Z\alpha}{9}\left(\frac{1}{3} - \frac{1}{6(1+\gamma)} + \frac{2}{\gamma} - \frac{3}{2\gamma - 1}\right)\,. \tag{S26}$$

The combined finite size and Bohr-Weisskopf correction to the shielding constant were calculated using formulas from ref. [42] (Results from this formula were found to be consistent with numerical results tabulated for low-Z ions in ref. [35]).

Table S5: Contributions to the shielding constant in $^9Be^{3+}$.

Contribution		$\sigma(^9Be^{3+})$	Following refs.
Leading value	all-order $Z\alpha$	0.000 071 165 01	[36–41]
Finite size		-0.000 000 000 16(3)	[42]
One-loop QED	all-order $(Z\alpha)$	0.000 000 000 23(13)	[35, 46]
Recoil	$\mathcal{O}\left(Z\alpha^2 \frac{m}{M}\right)$	-0.000 000 011 11(1)	[35–38]
Sum		0.000 071 153 97(14)	

Recoil corrections to the shielding constant were calculated using formulas from refs. [35, 43–45]

$$\begin{aligned} \sigma_{\rm rec} &= -\frac{\alpha Z\alpha}{3}\frac{m_e}{M_N}\left(1+\frac{g_N-1}{g_N}\right), \\ g_N &= \frac{M}{Zm_p}g_I\,. \end{aligned} \tag{S27}$$

The QED correction to the shielding constant was calculated to lowest order in $Z\alpha$ in ref. [46]. We estimate the QED contribution as given in Table S5 to all orders in $Z\alpha$, by extrapolating higher-order terms from ref. [35] to low Z. The uncertainty given in Table S5 takes into account the difference between our extrapolated all-order result and the $Z\alpha$ expansion result [46], as well as an estimation of uncalculated VP contributions [35].

Bound-electron g-factor

The theory of the bound-electron g-factor is very similar to the case of the $^3He^+$ ion [47]. The leading zero-loop g-factor contribution was first calculated for bound electrons in 1928 [48]. The contribution of QED Feynman diagrams with closed loops can be parametrized as the sum of the free electron's anomaly [16] and so-called binding corrections. For the light Be ion, binding corrections calculated in the framework of a perturbative expansion in the electron-nucleus interaction, with the expansion parameter being $Z\alpha$, are found to converge well. For the two-loop correction, already terms of order $(Z\alpha)^4$ and higher [49–53] are found to be smaller than 10^{-10}, as well as three-loop binding corrections of order $(Z\alpha)^2$. The higher-order contributions to the one-loop self-energy correction is based on the recent high-precision evaluation of these Feynman diagrams to all orders in $Z\alpha$ [54]. The Wichmann-Kroll contribution to the electric loop vacuum polarization correction [10] as well as the magnetic loop vacuum polarization correction [55] turn out to be too small to contribute at the given level of precision.

Unlike the case of the $^3He^+$ ion, the biggest uncertainty of theory contributions to the g_s- factor in $^9Be^{3+}$ originates from uncalculated higher-order QED corrections at the two-loop level [51]. It is slightly larger than the uncertainty of the finite nuclear size correction, which was calculated nuclear model independently using formulas and tabulated parameters from refs. [56, 57]. The uncertainty of the finite size contribution originates from the uncertainty of the nuclear root mean square radius as specified in ref. [58]. This means that a g-factor experiment with sufficient accuracy, combined with an independent mass measurement of the Be^{3+} ion, would allow for the direct and independent determination of the $r_{\rm rms}$ radius with an uncertainty which is comparable to the best available value [58].

3 Comparison plot data

The plotted data from Figure 4 of the main text is summarized in Table S7.

For the HFS of the hydrogen isotopes, we use the theoretical QED calculation from ref. [14] to evaluate the point-like contribution. The g-factor values, the fine-structure constant, which significantly contributes to the relative uncertainties of the zero-field splitting by about 3×10^{-10}, and the Rydberg constant are taken from CODATA [16]. Compared to our calculations for $^9Be^{3+}$, no contributions and associated uncertainties due to muonic vacuum polarization, hadronic vacuum polarization, and

Table S6: Contributions to the bound-electron g-factor in $^9Be^{3+}$. All digits given are significant.

Contribution		$^9Be^{3+}$	Following refs.
Dirac value		1.999 431 864 5	[10, 48]
Finite size		0.000 000 000 1	[56–62]
1-loop QED	$(Z\alpha)^0$	0.002 322 819 5	[16, 63]
	$(Z\alpha)^2$	0.000 000 329 8	[36–38, 64]
	$(Z\alpha)^4$	0.000 000 023 3	[49, 65]
	$(Z\alpha)^{5+}$ SE	0.000 000 001 1	[10, 66]
	$(Z\alpha)^{5+}$ VP-EL, Uehling	0.000 000 000 1	[10, 19, 67, 68]
2-loop QED	$(Z\alpha)^0$	-0.000 003 544 6	[16, 69, 70]
	$(Z\alpha)^2$	-0.000 000 000 5	[36–38, 64]
$\geq$ 3-loop QED	$(Z\alpha)^0$	0.000 000 029 5	[16, 71–75]
Recoil	$\frac{m}{M}$, all-order $Z\alpha$	0.000 000 051 9	[10, 61, 76–78]
Sum		2.001 751 574 7	

leading order recoil were included for H, D and T. Thus, for a better comparison with our results for $^9Be^{3+}$, the same calculation is applied to $^9Be^{3+}$ as well, see the Table.

For tritium, the experimental result of the ratio $\nu_{\mathrm{HFS}}(\mathrm{T})/\nu_{\mathrm{HFS}}(\mathrm{H})$, ref. [79] is combined with the hydrogen result, ref. [80].

For $^{209}Bi^{82+}$ the theoretical result from ref. [81] is rescaled with the new value of its nuclear magnetic moment, ref. [82].

Table S7: Data for Fig. 4. In the theoretical contribution of the hyperfine splittings, the contribution to the uncertainty of the experimental value of g_I is given if it is significant. The same applies to α and m_e. If no discrimination is made for the nuclear contribution, only the theoretical uncertainty is significant. The second line for $^9Be^{3+}$ uses theory calculations from ref. [14], which are used for the hydrogen isotopes H, D, T as well. For $^9Be^{3+}$ these do not include the same contributions as our calculations (thus the deviation of the values), and are only meant for the comparison with the hydrogen isotopes. For details, see text.

System	Exp. value	Theo. point nucl. value	Nucl. contrb.
HFS			
$^9Be^{3+}$ 1s	−12796971.342629(52) kHz [tw]	−12804791.6(3.5) kHz [tw]	7820.2(3.5) kHz
		−12804973.08(20) kHz [14]	8001.74(20) kHz
H 1s	1420405.751768(2) kHz [80]	$1420452.3711(14)(4)_{g_I}(4)_\alpha$ kHz [14]	−46.6193(15) kHz
D 1s	327384.3525222(17) kHz [83]	$327339.21932(33)(84)_{g_I}(10)_\alpha$ kHz [14]	45.13321(91) kHz
T 1s	1516701.4707745(74) kHz [79, 80]	$1516760.1094(15)(31)_{g_I}(5)_\alpha$ kHz [14]	−58.6386(34) kHz
$^3He^+$ 1s	−8665649.86577(26) kHz [47]	−8667379.5(1.3) kHz [47]	1729.7(1.3) kHz
$^{209}Bi^{82+}$ 1s	5085.03(1) meV [84]	$5137(3)_{g_I}$ meV [81]	−52(3) meV
$^9Be^+$ 2s	−625008.837044(12) kHz [28]	−625389.2(3.6) kHz [27]	380.4(3.6) kHz
$^6Li^+$ 2s	3001805.1(5.1) kHz [85]	$3002617.5(1.5)(1.4)_{g_I}$ kHz [14]	$-812.4(2.0)_{\mathrm{theo}}(5.1)_{\mathrm{exp}}$ kHz
$^7Li^+$ 2s	7926990(17) kHz [86]	$7929977.4(3.9)(1.3)_{g_I}$ kHz [14]	$-2987(4)_{\mathrm{theo}}(17)_{\mathrm{exp}}$ kHz
other			
µH Lamb shift	202.3706(23) meV [87]	206.0336(15) meV [87]	$-3.663(15)_{\mathrm{theo}}(23)_{\mathrm{exp}}$ meV
µD Lamb shift	202.8785(34) meV [88]	228.7766(10) meV [88]	$-25.8981(10)_{\mathrm{theo}}(34)_{\mathrm{exp}}$ meV
$g_s(^{118}Sn^{49+})$	1.910562059(9) [89]	1.91054733(30) [89]	$14.73(30) \times 10^{-6}$

References

1. Foreman-Mackey, D., Hogg, D. W., Lang, D. & Goodman, J. emcee: The MCMC Hammer. *Publications of the Astronomical Society of the Pacific* **125,** 306–312. doi:10.1086/670067 (2013).

2. Ketter, J., Eronen, T., Höcker, M., Streubel, S. & Blaum, K. First-order perturbative calculation of the frequency-shifts caused by static cylindrically-symmetric electric and magnetic imperfections of a Penning trap. *International Journal of Mass Spectrometry* **358,** 1–16. doi:10.1016/j.ijms.2013.10.005 (2014).

3. Heiße, F. *et al.* High-precision mass spectrometer for light ions. *Phys. Rev. A* **100,** 022518. doi:10.1103/PhysRevA.100.022518 (2019).

4. Schuh, M. *et al.* Image charge shift in high-precision Penning traps. *Phys. Rev. A* **100,** 023411. doi:10.1103/PhysRevA.100.023411 (2019).
5. Wineland, D. J. & Dehmelt, H. G. Principles of the stored ion calorimeter. *Journal of Applied Physics* **46,** 919–930. doi:10.1063/1.321602 (1975).
6. Moskovkin, D. L. & Shabaev, V. M. Zeeman effect of the hyperfine-structure levels in hydrogenlike ions. *Phys. Rev. A* **73,** 052506. doi:10.1103/PhysRevA.73.052506 (2006).
7. Stone, N. J. *Table of Nuclear Electric Quadrupole Moments* tech. rep. ATOMIC AND MOLECULAR PHYSICS (International Atomic Energy Agency (IAEA), 2021), 68.
8. Pearson, K. Note on Regression and Inheritance in the Case of Two Parents. *Proceedings of the Royal Society of London Series I* **58,** 240–242 (1895).
9. Stone, N. *Table of recommended nuclear magnetic dipole moments* tech. rep. (International Atomic Energy Agency, 2019).
10. Beier, T. The g_j factor of a bound electron and the hyperfine structure splitting in hydrogenlike ions. *Phys. Rep.* **339,** 79–213 (2000).
11. Karshenboim, S. G. Leading logarithmic corrections and uncertainty of Muonium hyperfine splitting calculations. *Zeitschrift für Physik D Atoms, Molecules and Clusters* **36,** 11–15. doi:10.1007/BF01437414 (1996).
12. Bodwin, G. T., Yennie, D. R. & Gregorio, M. A. Recoil effects in the hyperfine structure of QED bound states. *Rev. Mod. Phys.* **57,** 723–782. doi:10.1103/RevModPhys.57.723 (1985).
13. Bodwin, G. T. & Yennie, D. R. Some recoil corrections to the hydrogen hyperfine splitting. *Phys. Rev. D* **37,** 498–523. doi:10.1103/PhysRevD.37.498 (1988).
14. Pachucki, K., Patkóš, V. & Yerokhin, V. A. Hyperfine splitting in ^{6,7}Li^{+}. *Phys. Rev. A* **108,** 052802. doi:10.1103/PhysRevA.108.052802 (2023).
15. Kinoshita, T. NRQED Approach to the Hyperfine Structure of the Muonium Ground State. *High Energy Physics - Phenomenology (hep-ph).* arXiv:hep-ph/9808351v1 (1998).
16. Tiesinga, E., Mohr, P. J., Newell, D. B. & Taylor, B. N. CODATA recommended values of the fundamental physical constants: 2018. *Rev. Mod. Phys.* **93,** 025010. doi:10.1103/RevModPhys.93.025010 (2021).
17. Yerokhin, V. A. Hyperfine structure of Li and Be^{+}. *Phys. Rev. A* **78,** 012513. doi:10.1103/PhysRevA.78.012513 (2008).
18. J. Heiland Hoyo & B. Sikora. *Hadronic vacuum polarization correction to the hyperfine splitting in hydrogenlike ions* in preparation.
19. Peskin, M. E. & Schroeder, D. V. *An Introduction to Quantum Field Theory* (Westview Press, 1995).
20. Shabaev, V. M. in *Precision Physics of Simple Atomic Systems* (eds Karshenboim, S. G. & Smirnov, V. B.) 97–113 (Springer-Verlag, Berlin Heidelberg, 2003).
21. Karshenboim, S. G., Ivanov, V. G. & Shabaev, V. M. Polarization of vacuum in a hydrogen-like relativistic atom: Hyperfine structure. *Journal of Experimental and Theoretical Physics* **90,** 59–65. doi:10.1134/1.559094 (2000).
22. Schneider, S. M., Greiner, W. & Soff, G. Vacuum-polarization contribution to the hyperfine-structure splitting of hydrogenlike atoms. *Phys. Rev. A* **50,** 118–122. doi:10.1103/PhysRevA.50.118 (1994).
23. A. V. Volotka. *High-precision QED calculations of the hyperfine structure in hydrogen and transition rates in multicharged ions* PhD thesis (Technische Universität Dresden, 2006).
24. Karshenboim, S. G. & Ivanov, V. G. Hyperfine structure of the ground and first excited states in light hydrogen-like atoms and high-precision tests of QED. *The European Physical Journal D - Atomic, Molecular, Optical and Plasma Physics* **19,** 13–23. doi:10.1140/epjd/e20020050 (2002).

25. Shabaev, V. M. Hyperfine structure of hydrogen-like ions. *Journal of Physics B: Atomic, Molecular and Optical Physics* **27,** 5825. doi:10.1088/0953-4075/27/24/006 (1994).
26. Karshenboim, S. G. Nuclear structure-dependent radiative corrections to the hydrogen hyperfine splitting. *Physics Letters A* **225,** 97–106. doi:https://doi.org/10.1016/S0375-9601(96)00861-4 (1997).
27. Puchalski, M. & Pachucki, K. Ground-state hyperfine splitting in the Be^+ ion. *Phys. Rev. A* **89,** 032510. doi:10.1103/PhysRevA.89.032510 (2014).
28. Shiga, N., Itano, W. M. & Bollinger, J. J. Diamagnetic correction to the $^9Be^+$ ground-state hyperfine constant. *Phys. Rev. A* **84,** 012510. doi:10.1103/PhysRevA.84.012510 (2011).
29. Shabaev, V. M., Artemyev, A. N., Yerokhin, V. A., Zherebtsov, O. M. & Soff, G. Towards a Test of QED in Investigations of the Hyperfine Splitting in Heavy Ions. *Phys. Rev. Lett.* **86,** 3959–3962. doi:10.1103/PhysRevLett.86.3959 (2001).
30. Pachucki, K. Nuclear recoil correction to the hyperfine splitting in atomic systems. *Phys. Rev. A* **106,** 022802. doi:10.1103/PhysRevA.106.022802 (2022).
31. Patoary, A. S. M. & Oreshkina, N. S. Finite nuclear size effect to the fine structure of heavy muonic atoms. *The European Physical Journal D* **72,** 54. doi:10.1140/epjd/e2018-80545-9 (2018).
32. Volotka, A. V., Shabaev, V. M., Plunien, G. & Soff, G. Zemach and magnetic radius of the proton from the hyperfine splitting in hydrogen. *The European Physical Journal D - Atomic, Molecular, Optical and Plasma Physics* **33,** 23–27. doi:10.1140/epjd/e2005-00025-9 (2005).
33. Feynman, R., Leighton, R. & Sands, M. *he Feynman Lectures on Physics, Vol. III: The New Millennium Edition: Quantum Mechanics* (Basic Books, 2011).
34. Varentsova, A. *et al.* Third-order Zeeman effect in highly charged ions. *Nuclear Instruments and Methods in Physics Research Section B: Beam Interactions with Materials and Atoms* **408.** Proceedings of the 18th International Conference on the Physics of Highly Charged Ions (HCI-2016), Kielce, Poland, 11-16 September 2016, 80–83. doi:https://doi.org/10.1016/j.nimb.2017.05.040 (2017).
35. Yerokhin, V. A., Pachucki, K., Harman, Z. & Keitel, C. H. QED calculation of the nuclear magnetic shielding for hydrogenlike ions. *Phys. Rev. A* **85,** 022512. doi:10.1103/PhysRevA.85.022512 (2012).
36. Grotch, H. & Hegstrom, R. A. Hydrogenic Atoms in a Magnetic Field. *Phys. Rev. A* **4,** 59–69. doi:10.1103/PhysRevA.4.59 (1971).
37. Close, F. E. & Osborn, H. Relativistic extension of the electromagnetic current for composite systems. *Phys. Lett. B* **34,** 400–404 (1971).
38. Faustov, R. Magnetic moment of the hydrogen atom. *Physics Letters B* **33,** 422–424. doi:https://doi.org/10.1016/0370-2693(70)90621-0 (1970).
39. MOORE, E. A. Relativistic chemical shielding: formally exact solutions for one—electron atoms of maximum total angular momentum for any principal quantum number. *Molecular Physics* **97,** 375–380. doi:10.1080/00268979909482838 (1999).
40. Pyper, N. Relativistic theory of nuclear shielding in one-electron atoms 1. Theoretical foundations and first-order terms. *Molecular Physics* **97,** 381–390. doi:10.1080/00268979909482839 (1999).
41. Pyper, N. C. & Zhang, Z. C. Relativistic theory of nuclear shielding in one-electron atoms 2. Analytical and numerical results. *Molecular Physics* **97,** 391–413. doi:10.1080/00268979909482840 (1999).
42. Pachucki, K. Nuclear mass and size corrections to the magnetic shielding. *Phys. Rev. A* **108,** 062806. doi:10.1103/PhysRevA.108.062806 (2023).
43. Eides, M. I. & Grotch, H. Gyromagnetic Ratios of Bound Particles. *Ann. Phys.* **260,** 191–200 (1997).

44. Pachucki, K. Nuclear mass correction to the magnetic interaction of atomic systems. *Phys. Rev. A* **78,** 012504 (2008).

45. Pachucki, K., Patkóš, V. & Yerokhin, V. A. Accurate determination of 6,7Li nuclear magnetic moments. *Physics Letters B,* 138189. doi:https://doi.org/10.1016/j.physletb.2023.138189 (2023).

46. Wehrli, D., Spyszkiewicz-Kaczmarek, A., Puchalski, M. & Pachucki, K. QED Effect on the Nuclear Magnetic Shielding of ^{3}He. *Phys. Rev. Lett.* **127,** 263001. doi:10.1103/PhysRevLett.127.263001 (2021).

47. Schneider, A. *et al.* Direct measurement of the 3He+ magnetic moments. *Nature* **606,** 878–883. doi:10.1038/s41586-022-04761-7 (2022).

48. Breit, G. The Magnetic Moment of the Electron. *Nature* **122,** 649–649 (1928).

49. Pachucki, K., Czarnecki, A., Jentschura, U. D. & Yerokhin, V. A. Complete two-loop correction to the bound-electron g factor. *Phys. Rev. A* **72,** 022108 (2005).

50. Czarnecki, A. & Szafron, R. Light-by-light scattering in the Lamb shift and the bound electron g factor. *Phys. Rev. A* **94,** 060501 (2016).

51. Czarnecki, A., Dowling, M., Piclum, J. & Szafron, R. Two-Loop Binding Corrections to the Electron Gyromagnetic Factor. *Phys. Rev. Lett.* **120,** 043203 (2018).

52. Czarnecki, A., Piclum, J. & Szafron, R. Logarithmically enhanced Euler-Heisenberg Lagrangian contribution to the electron gyromagnetic factor. *Phys. Rev. A* **102,** 050801. doi:10.1103/PhysRevA.102.050801 (2020).

53. Yerokhin, V. A. & Harman, Z. Two-loop QED corrections with closed fermion loops for the bound-electron g factor. *Phys. Rev. A* **88,** 042502 (2013).

54. Yerokhin, V. A. & Harman, Z. One-loop electron self-energy for the bound-electron g factor. *Phys. Rev. A* **95,** 060501 (2017).

55. Lee, R. N., Milstein, A. I., Terekhov, I. S. & Karshenboim, S. G. Virtual light-by-light scattering and the g factor of a bound electron. *Phys. Rev. A* **71,** 052501 (2005).

56. Yerokhin, V. A., Keitel, C. H. & Harman, Z. Nuclear-size self-energy and vacuum-polarization corrections to the bound-electron g factor. *J. Phys. B* **46,** 245002 (2013).

57. Zatorski, J., Oreshkina, N. S., Keitel, C. H. & Harman, Z. Nuclear Shape Effect on the g Factor of Hydrogenlike Ions. *Phys. Rev. Lett.* **108,** 063005 (2012).

58. Angeli, I. & Marinova, K. P. Table of experimental nuclear ground state charge radii: An update. *At. Data Nucl. Data Tables* **99,** 69–95 (2013).

59. Trouyet, Y. & Sikora, B. *Finite nuclear size correction to the bound-electron g-factor for the determination of nuclear radii* in preparation.

60. Moutet, M. & Sikora, B. *Finite size corrections to the hyperfine splitting* in preparation.

61. Fischer, C. F., Gaigalas, G., Jönsson, P. & Bieroń, J. GRASP2018 – A Fortran 95 version of the General Relativistic Atomic Structure Package. *Comput. Phys. Commun.* **237,** 184–187. doi:https://doi.org/10.1016/j.cpc.2018.10.032 (2019).

62. Karshenboim, S. G. & Ivanov, V. G. Finite-nuclear-size contribution to the g factor of a bound electron: Higher-order effects. *Phys. Rev. A* **97,** 022506. doi:10.1103/PhysRevA.97.022506 (2018).

63. Schwinger, J. On Quantum-Electrodynamics and the Magnetic Moment of the Electron. *Phys. Rev.* **73,** 416–417 (1948).

64. A. Czarnecki, K. Melnikov & A. Yelkhovsky. Anomalous magnetic moment of a bound electron. *Phys. Rev. A* **63,** 012509 (2000).

65. Pachucki, K., Jentschura, U. D. & Yerokhin, V. A. Nonrelativistic QED Approach to the Bound-Electron g Factor. *Phys. Rev. Lett.* **93,** 150401 (2004).

66. Yerokhin, V. A., Indelicato, P. & Shabaev, V. M. Evaluation of the self-energy correction to the g factor of S states in H-like ions. *Phys. Rev. A* **69,** 052503 (2004).

67. Karshenboim, S. G., Ivanov, V. G. & Shabaev, V. M. Vacuum Polarization in a Hydrogen-like Relativistic Atom: g Factor of a Bound Electron. *J. Exp. Theor. Phys. Lett.* **93,** 477 (2001).

68. Karshenboim, S. G. Precision physics of simple atoms: QED tests, nuclear structure and fundamental constants. *Physics Reports* **422,** 1–63. doi:https://doi.org/10.1016/j.physrep.2005.08.008 (2005).

69. Petermann, A. Fourth order magnetic moment of the electron. *Helv. Phys. Acta* **30,** 407–408 (1957).

70. Sommerfield, C. M. The Magnetic Moment of the Electron. *Ann. Phys.* **5,** 26–57 (1958).

71. Laporta, S. & Remiddi, E. The analytical value of the electron $g-2$ at order α^3 in QED. *Phys. Lett. B* **379,** 283–291 (1996).

72. Aoyama, T., Hayakawa, M., Kinoshita, T. & Nio, M. Revised Value of the Eighth-Order Contribution to the Electron $g-2$. *Phys. Rev. Lett.* **99,** 110406 (2007).

73. Aoyama, T., Hayakawa, M., Kinoshita, T. & Nio, M. Tenth-Order QED Contribution to the Electron $g-2$ and an Improved Value of the Fine Structure Constant. *Phys. Rev. Lett.* **109,** 111807 (2012).

74. Laporta, S. High-precision calculation of the 4-loop contribution to the electron $g-2$ in QED. *Phys. Lett. B* **772,** 232–238 (2017).

75. Aoyama, T., Hayakawa, M., Kinoshita, T. & Nio, M. Erratum: Tenth-order electron anomalous magnetic moment: Contribution of diagrams without closed lepton loops [Phys. Rev. D 91, 033006 (2015)]. *Phys. Rev. D* **96,** 019901(E) (2017).

76. Shabaev, V. M. & Yerokhin, V. A. Recoil Correction to the Bound-Electron g Factor in H-Like Atoms to All Orders in αZ. *Phys. Rev. Lett.* **88,** 091801 (2002).

77. Wang, M., Huang, W., Kondev, F., Audi, G. & Naimi, S. The AME 2020 atomic mass evaluation (II). Tables, graphs and references*. *Chinese Physics C* **45,** 030003. doi:10.1088/1674-1137/abddaf (2021).

78. Kramida, A., Ralchenko, Y., J. Reader & the NIST ASD Team. *NIST Atomic Spectra Database (ver. 5.9), [Online]* National Institute of Standards and Technology, Gaithersburg, MD. 2021.

79. Mathur, B. S., Crampton, S. B., Kleppner, D. & Ramsey, N. F. Hyperfine Separation of Tritium. *Phys. Rev.* **158,** 14–17. doi:10.1103/PhysRev.158.14 (1967).

80. Hellwig, H. *et al.* Measurement of the Unperturbed Hydrogen Hyperfine Transition Frequency. *IEEE Transactions on Instrumentation and Measurement* **19,** 200–209. doi:10.1109/TIM.1970.4313902 (1970).

81. Shabaev, V. M. *et al.* Calculation of the hyperfine structure of heavy H and Li like ions. *Hyperfine Interactions* **127,** 279–286. doi:10.1023/A:1012616322709 (2000).

82. Skripnikov, L. V. *et al.* New Nuclear Magnetic Moment of ^{209}Bi: Resolving the Bismuth Hyperfine Puzzle. *Phys. Rev. Lett.* **120,** 093001. doi:10.1103/PhysRevLett.120.093001 (2018).

83. Wineland, D. J. & Ramsey, N. F. Atomic Deuterium Maser. *Phys. Rev. A* **5,** 821–837. doi:10.1103/PhysRevA.5.821 (1972).

84. Ullmann, J. *et al.* High precision hyperfine measurements in Bismuth challenge bound-state strong-field QED. *Nature Communications* **8,** 15484 (2017).

85. Sun, W. *et al.* Measurement of Hyperfine Structure and the Zemach Radius in ^{6}Li$^+$ Using Optical Ramsey Technique. *Phys. Rev. Lett.* **131,** 103002. doi:10.1103/PhysRevLett.131.103002 (2023).

86. Guan, H. *et al.* Probing atomic and nuclear properties with precision spectroscopy of fine and hyperfine structures in the ^{7}Li$^+$ ion. *Phys. Rev. A* **102,** 030801. doi:10.1103/PhysRevA.102.030801 (2020).

87. Antognini, A. *et al.* Proton Structure from the Measurement of 2S-2P Transition Frequencies of Muonic Hydrogen. *Science* **339,** 417–420. doi:10.1126/science.1230016 (2013).

88. Pohl, R. *et al.* Laser spectroscopy of muonic deuterium. *Science* **353,** 669–673. doi:10.1126/science.aaf2468 (2016).

89. Morgner, J. *et al.* Stringent test of QED with hydrogen-like tin. *Nature* **622,** 53–57. doi:10.1038/s41586-023-06453-2 (2023).
